# Standing Practice In Rehabilitation Early after Stroke (SPIRES): a functional standing frame programme (prolonged standing and repeated sit to stand) to improve function and quality of life and reduce neuromuscular impairment in people with severe sub-acute stroke—a protocol for a feasibility randomised controlled trial

**DOI:** 10.1186/s40814-018-0254-z

**Published:** 2018-03-23

**Authors:** Angie Logan, Jennifer Freeman, Bridie Kent, Jillian Pooler, Siobhan Creanor, Jane Vickery, Doyo Enki, Andrew Barton, Jonathan Marsden

**Affiliations:** 1 Faculty of Health and Human Sciences, School of Health Professions, Peninsula Allied Health Centre, Plymouth University, Derriford Rd, Plymouth, PL6 8BH UK; 2 School of Nursing and Midwifery, Plymouth University, Room 405, Rolle Building, Drake Circus, Plymouth, Devon PL4 8AA UK; 3 Peninsula Schools of Medicine and Dentistry, Rooms 14 & 15, ITTC Building Research Way, Plymouth, PL6 8BU UK; 4 Peninsula Clinical Trials Unit (PenCTU), Plymouth University Peninsula Schools of Medicine and Dentistry, Room N16, Plymouth Science Park, Plymouth, PL6 8BX UK; 5 Medical Statistics, Peninsula Schools of Medicine and Dentistry, Room N15, Plymouth Science Park, Plymouth, PL6 8BX UK; 6 National Institute for Health Research, Research Design Service, Peninsula Schools of Medicine and Dentistry, ITTC Building, Plymouth Science Park, Plymouth, PL6 8BX UK

**Keywords:** Stroke, Supported standing, Neuromuscular impairment, Physiotherapy, Function, Feasibility randomised controlled trial, Early mobilisation

## Abstract

**Background:**

The most common physical deficit caused by a stroke is muscle weakness which limits a person’s mobility. Mobility encompasses activities necessary for daily functioning: getting in and out bed, on/off toilet, sitting, standing and walking. These activities are significantly affected in people with severe stroke who typically spend most of their time in bed or a chair and are immobile. Immobility is primarily caused by neurological damage but exacerbated by secondary changes in musculoskeletal and cardiorespiratory systems. These secondary changes can theoretically be prevented or minimised by early mobilisation, in this case standing up early post-stroke.

Standing up early post-stroke has been identified as an important priority for people who have suffered a severe stroke. However, trials of prolonged passive standing have not demonstrated any functional improvements. Conversely, task-specific training such as repeated sit-to-stand has demonstrated positive functional benefits. This feasibility trial combines prolonged standing and task-specific strength training with the aim of determining whether this novel combination of physiotherapy interventions is feasible for people with severe stroke as well as the overall feasibility of delivering the trial.

**Methods/design:**

This is a pragmatic multi-centre parallel single-blinded two-armed feasibility randomised controlled trial. Fifty people with a diagnosis of severe stroke will be randomly allocated to either the functional standing frame programme or usual physiotherapy. All patient participants will be assessed at baseline and followed up at 3 weeks, then 3, 6 and 12 months post-randomisation. Trial objectives are to determine the feasibility according to the following indicators:: (i) *Process*: recruitment and retention rate, ability to consent, eligibility criteria, willingness/ability of physiotherapists to recruit, willingness of patients to be randomised, and acceptability of the intervention; (ii) *Resource*: burden and potential costs; (iii) *Management*: treatment fidelity, participant adherence, acceptability and completeness of outcome measures, impact and management or orthostatic hypotension; and (iv) *Safety*: number and nature of adverse and serious adverse events.

**Discussion:**

The functional standing frame programme addresses a key concern for people who have suffered a severe stroke. However, several uncertainties exist which need to be understood prior to progressing to a full-scale trial, including acceptability and tolerance of the functional standing frame programme intervention and practicality of the trial procedures. This feasibility trial will provide important insights to resolve these uncertainties.

**Trial registration:**

International Standard Randomised Controlled Trial Number ISRCTN15412695. Registration on 19 December 2016.

**Electronic supplementary material:**

The online version of this article (10.1186/s40814-018-0254-z) contains supplementary material, which is available to authorized users.

## Background

Stroke is a sudden and devastating illness affecting over 100,000 people per annum in the United Kingdom (UK) [53]. Current government and clinical guidelines recommend that following diagnosis of a stroke, people are admitted to an Acute Stroke Unit to receive stroke specialist multi-disciplinary care. This reduces mortality and improves functional outcomes [40] compared to standard non-specialist care. The UK stroke pathway [44] and National Clinical Guidelines [34] advocate that people with mild or moderate strokes (i.e. modified Rankin Scale (mRS) 1–3; able to transfer independently or with the help of one person with/without equipment) are discharged from the Acute Stroke Unit to the Early Supported Discharge service, which provides specialist stroke rehabilitation in a home-based setting. People with severe strokes (mRS 4–5; requiring assistance of two people with/without equipment) are transferred from the Acute Stroke Unit to a specialist Stroke Rehabilitation Unit for early sub-acute rehabilitation usually within 7 days. The early sub-acute phase spans from 7 days to 3 months [6]), with average time from stroke onset to admission on Stroke Rehabilitation Unit 6 days (1 to 37 days) [13]. Some Early Supported Discharge services in the UK admit people with a mRS of 4, but this is not the standard care nationally.

The implementation of Early Supported Discharge has caused a change in patient caseload nationally for Stroke Rehabilitation Units [54] resulting in the majority of people admitted to Stroke Rehabilitation Units having complex needs and severe deficits. This change in caseload necessitates the design and evaluation of interventions for patients in Stroke Rehabilitation Units that target people with more severe deficits. The most common physical deficit caused by stroke is motor impairment, seen in approximately 80% of people [70]. It is the single most disabling factor in terms of limiting a person’s mobility, their ability to participate in activities of daily living (ADL) and to live independently [67]. Mobility encompasses a wide range of activities necessary for daily functioning: moving in bed, getting in/out of bed, on/off toilet, sitting out of bed, standing and walking [34]. These activities are particularly affected in the 15.5% of people with severe stroke [54] who require the assistance of two people to undertake ADL, may need equipment to aid transfers and are unable to sit unsupported, stand or walk [27]. They typically spend much of their time in bed and are dependent on a wheelchair/specialist seating when they sit out of bed [57].

Although immobility post-stroke is primarily caused by neurological damage, it can be exacerbated by other factors such as muscle wasting [56], reduced muscle length, increased muscle stiffness [47], joint contracture [26] and orthostatic hypotension. Orthostatic Hypotension (OH) is a sudden drop in blood pressure when moving from lying to standing, leading to symptoms of feeling faint, generalised weakness, cognitive slowing, and gradual or sudden loss of consciousness [38] and can, therefore, limit standing time. It can affect over 50% of people post-stroke [37] and should be addressed using non-pharmacological or pharmacological interventions, or a combination of the two [49, 64]. Secondary changes in the musculoskeletal and cardiorespiratory systems can theoretically be prevented or minimised by early mobilisation [65] especially standing [46]; prevention of these secondary changes underpins the rationale for the current trial.

Several randomised controlled trials (RCTs) have reported mixed outcomes of early mobilisation post-stroke. Less favourable outcomes occurred when early mobilisation was instigated *very* early (within the first 24 h post-stroke) [62] compared to a reduction in complications when instigated early (≥ 24 h post-stroke) [17]. Early mobilisation is associated with increased independence in ADL and a faster return to walking [15]. However, these trials all had different primary end-points (Functional Independence Measure; Modified Rankin; incidence of severe complications during hospitalisation) which makes direct comparison of effectiveness of early mobilisation interventions difficult. Whilst early mobilisation is deemed to be safe [5, 15, 17, 62], uncertainties have been identified with regard to dose and frequency. The AVERT Trial Collaborative Group suggest that shorter, more frequent mobilisation is preferable, and the latest Royal College of Physicians Guidelines have incorporated this, recommending patients “accumulate” at least 45 min daily. However, the main limitation of these studies is that they specify neither the time spent mobilising nor the intensity, content and frequency of therapy. Therefore, it is possible in previous work that, other than hoists, no specialised equipment was used to promote recovery for people with severe stroke, and active sitting constituted early mobilisation with no opportunity to stand. This is aligned with current clinical practice in Cornwall and Devon (where this research is being led) where standing is not routinely implemented as part of sub-acute inpatient rehabilitation for people who have suffered a severe stroke.

A motorised standing frame can safely assist people with severe stroke into a supported standing posture. Suggested benefits of supported standing include stretching contracted muscles, decreasing spasticity, strengthening muscles, improving bladder and bowel function, relieving pressure areas and reducing OH [2, 19, 31, 35, 45, 69]. Evidence from people with spinal cord injury, multiple sclerosis, stroke and traumatic brain injury [2, 46, 60] indicates that the aforementioned benefits can be observed with 30 min of regular standing. However, a systematic review highlighted variation in duration (20 to 60 min) and frequency from three times per week (median 5 days per week), with dose dependent on length of inpatient say and participant recovery [46].

In preparation for this feasibility trial, discussions with people who had suffered a stroke and their relatives identified that standing up early after a stroke was important, relevant and meaningful for them and this formed the basis of the research question and subsequent trial design/development. Opportunities to stand for people who have suffered a severe stroke are limited due to significant disability; thus, they are reliant on physical assistance from mechanical equipment such as a standing frame.

Standing frames are not routinely used in sub-acute inpatient stroke rehabilitation as part of a postural management programme and never issued on discharge from Stroke Rehabilitation Units. A systematic review [46] identified a paucity of robust evidence for the use of standing frames in people with stroke. A previous RCT found no difference in functional outcomes in people with a sub-acute severe stroke who did or did not use standing frames for 14 consecutive sessions [1]. The lack of functional improvement may be due to the duration and intensity of treatment, as well as the sensitivity of the outcome measures used (Rivermead Mobility Index and Barthel Index). Additionally, participants undertook prolonged passive standing only and the addition of task-specific training may have improved functional outcomes. Task-specific training is based on the fundamental principle that repeated practice of functionally relevant tasks is the best way to learn [3, 23]. Tasks such as sit-to-stand have been shown to produce functional benefits post-stroke, and patients who receive task-specific training are more likely to improve their function and sustain these improvements than patients receiving usual care [23, 63]. The combination of prolonged standing and task-specific strength training underpins the rationale for the functional standing frame programme used in this feasibility trial.

The functional standing frame intervention combines two physiotherapy interventions that have separately been evaluated and reported in the literature: prolonged standing and task-specific strength training. Currently, it is not known whether this novel combination of physiotherapy for people with severe stroke is effective in everyday clinical practice, and the United Kingdom Medical Research Council provides guidance on how complex interventions can be developed and evaluated [42]. A key component of the evaluation of the complex intervention is the acceptability among people with severe stroke. Currently, it is not known whether the functional standing frame programme would be tolerated or acceptable for people with severe stroke, at this early stage of their rehabilitation continuum. The Medical Research Council guidance explicitly recommends an early phase of assessing feasibility prior to a full definitive main trial [43] and recognises the value of using both quantitative and qualitative methods concurrently. The use of qualitative research within feasibility RCTs is becoming increasingly common [48]. This feasibility randomised controlled trial facilitates the evaluation of acceptability, compliance and delivery of the intervention; trial practices, processes and design; and recruitment and refusal and retention rates. This links to the trial objectives and will inform the conduct and design of the anticipated definitive main trial to facilitate successful, effective and confident delivery.

Qualitative research will be embedded in this feasibility trial and used in tandem with quantitative methods to provide insights into the intervention and trial processes.

## Aims

The *primary aim* of this trial is to establish whether a RCT of a functional standing frame programme in people with severe stroke in an inpatient sub-acute stroke rehabilitation setting is feasible.

The *secondary aim* is to explore the experience of this functional standing frame programme on the individual’s daily life, as well as the experience of being recruited and randomised to the trial, from the perspective of both the person with stroke and their relative/carer, using qualitative methods.

## Objectives

Trial-specific objectives are to evaluate the feasibility according to the following indicators:

### Process

#### Recruitment rate

The recruitment rate will be defined as the number of participants recruited per month. This information was recorded in the study log at each site.

#### Retention rate

The retention rate will be calculated by dividing the number of participants, who completed data collection at T2 (post-intervention), T3 (3-month follow-up), T4 (6-month follow-up) and T5 (12-month follow-up) by the number of participants who completed data collection at T1.

#### Ability to consent

The ability of patient participants to consent will be measured by the number of participants who provided informed consent and the number of consultee declarations [16]. Additionally, incidence of cognitive and communication impairments will be measured from the Screening and Post-Screening and Assessor Case Report Forms.

#### Consent rate

The consent rate will be calculated by dividing the number of individuals who met inclusion criteria, by the number who consented to participate in the trial. Reasons why eligible individuals are not interested in participating will be recorded by the PI/recruiting therapist in the approach/screening log.

#### Eligibility criteria

The suitability and feasibility of eligibility criteria will be determined by reviewing reasons for exclusion documented in the Approach/Screening Log and Screening and Post-Screening Case Report Forms, and reviewing characteristics of recruited patient participants documented in the Screening and Post-Screening Case Report Forms

#### Willingness/ability of physiotherapists

The willingness or ability of physiotherapists to recruit will be measured by subtracting the number of patient participants screened and approached from the number of admissions documented on the Approach/Screening Log

#### Willingness of patients to be randomised

The willingness of patient participants to be randomised will be measured by the recruitment rate, and number of participants who refused as documented on the Approach/Screening Log

#### Acceptability of the intervention

Acceptability of the intervention among patients, relatives and physiotherapists will be measured by number of withdrawals (requested by patient, relative or healthcare professional), all of the points about under the heading “Process” in addition to qualitative data collected via semi-structured interviews with patient participants, their relatives and physiotherapists and a focus group with physiotherapists.

#### Determining usual physiotherapy

Determining usual physiotherapy management for people who have had a severe stroke receiving inpatient early sub-acute stroke rehabilitation will be captured by the Control Group Case Report Forms.

#### Sample size estimates

Sample size estimates, together with existing literature and standard deviation of the outcome measures, will help to inform power calculations for subsequent trials.

### Resource

#### Burden

Patient participant and physiotherapist burden will be measured by number of patient participants refusing therapy sessions and follow-up assessments. This will also be explored in semi-structured interviews with patients, their relatives and physiotherapists, focus group with physiotherapists, and field notes from blinded assessor.

#### Cost effectiveness

Estimates of resource use and related costs for the delivery of Standing Practice In Rehabilitation Early after Stroke (SPIRES) will be measured through semi-structured interviews exploring time required for preparation for functional standing programme session. Duration of functional standing frame programme group session (total minutes) will be captured in the Case Report Form and will be compared with the duration (total minutes) of the usual physiotherapy (control) group.

### Management

#### Fidelity

Intervention fidelity, defined as adherent delivery of the intervention, will be evaluated using a trial-specific SPIRES checklist that outlined all components of the functional standing frame programme intervention, and usual physiotherapy control group to be completed by an independent observer (e.g. physiotherapist checked blood pressure, demonstrated, ensure foot sensors in situ and positioned safely, position participant in frame etc.).

#### Participant adherence

The tolerance/adherence of the functional standing frame programme to people who have had a severe stroke will be measured by tracking (i) total number of sessions completed; (ii) total number of minutes standing; (iii) total number of sit to stand repetitions; (iv) enjoyment; (v) effort; (vi) fatigue; and (vii) reasons for non-completion of sessions, as documented by treating physiotherapists in the Case Report Form.

#### Feasibility of outcome measures

The feasibility of the proposed outcome measures will be measured by the number of primary and secondary outcome measures completed and the ability to detect change in this patient group with severe mobility impairment.

#### Orthostatic Hypotension protocol

The feasibility and acceptability of the Orthostatic Hypotension protocol will be measured by the incidence of orthostatic hypotension, the number of incomplete sessions due to orthostatic hypotension and the number of participants who received pharmacological or non-pharmacological (abdominal binders) treatment.

### Safety

#### Intervention

Safety of the intervention will be measured by the number of adverse events and serious adverse events that occur during the SPIRES intervention period (e.g. falls, skin damage, infection, hospital admission, death). The treating physiotherapists are responsible for documenting any adverse events or serious adverse events.

#### Data collection

Safety during data collection will be assessed by the number of adverse events or serious adverse events that occurred during the follow-up period. The blinded assessors are responsible for recoding any adverse events that occurred during data collection.

More specifically the *objectives* related to the qualitative evaluation are to:

- Explore means by which the trial procedures (timing and mode of participant recruitment, information provision, methods of data collection for example timing and content of outcome measures) can be refined to maximise recruitment, retention and acceptability in the definitive trial
- Explore patient participants’ experience of the functional standing frame programme
- Explore patient participants’ experience of being randomised
- Explore patient participants’ reasons for, and experience of, withdrawing from the trial
- Explore relatives’ influence in participants’ decision to consent to participate, remain in the trial or provide assent for their relative
- Explore physiotherapists’ attitudes, thoughts and feelings of implementing the intervention and whether they perceive a subsequent RCT to be achievable
- Explore physiotherapists’ attitudes, thoughts and feeling of the trial documentation and processes.

### Trial design

**Fig. 1:**
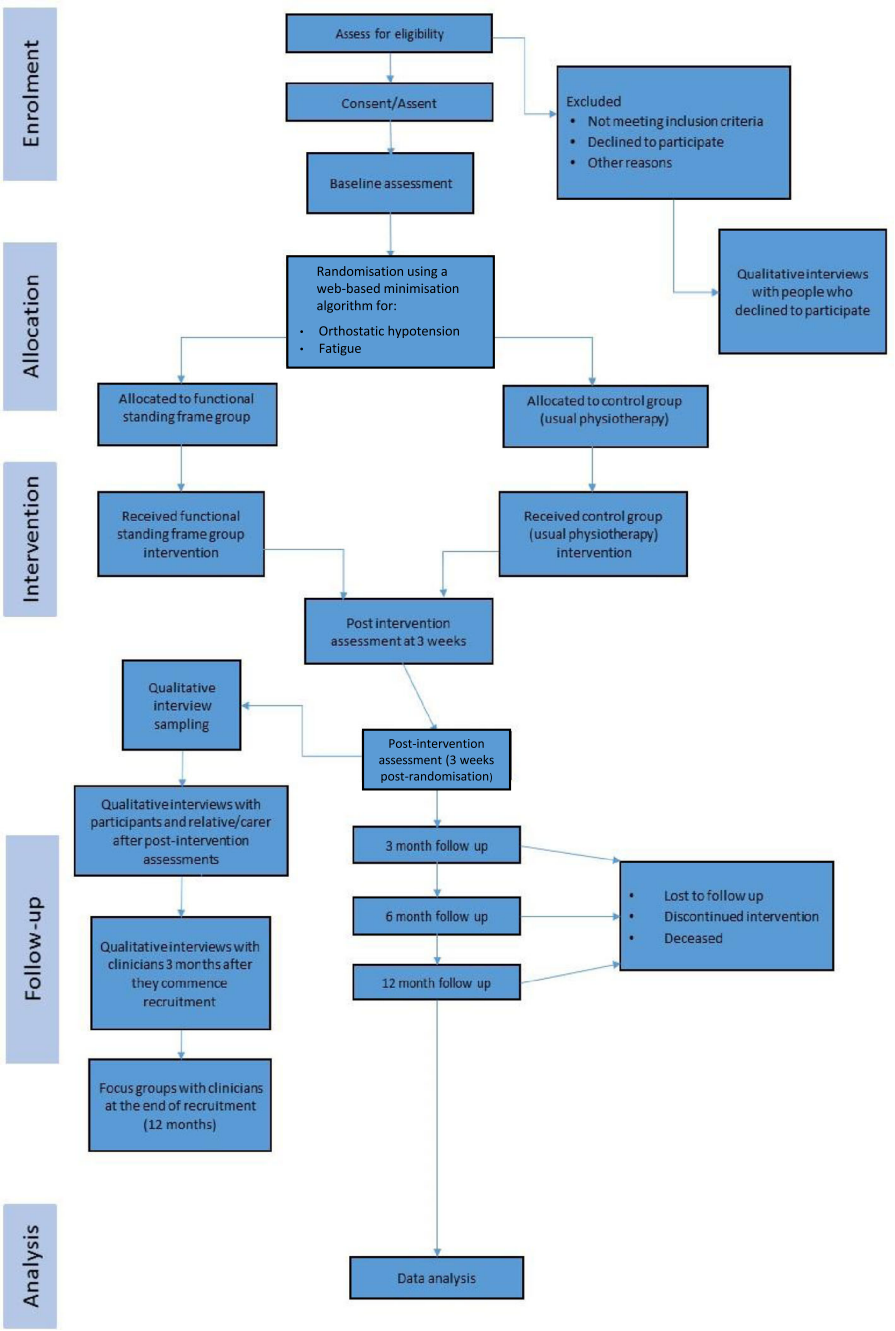
Trial flow chart. This figure represents the trial processes from assessing eligibility to data analysis

This is a pragmatic, multi-centre, parallel single-blinded two-armed feasibility RCT (Fig. 1).

## Methods: participants, intervention and outcomes

### Trial setting

Three healthcare sites (with four Stroke Rehabilitation Units) will be involved in the trial, which is based in two counties in the South West Peninsula of England. A full list of trial sites is available via https://www.plymouth.ac.uk/research/spires.

### Eligibility criteria

#### Inclusion criteria for quantitative methods

The trial population will comprise individuals with a confirmed clinical diagnosis of new (first or recurrent) severe stroke, cerebral haemorrhage or infarct confirmed by consultant or computed tomography (CT) scan and leading to admission to the Stroke Rehabilitation Unit. Participants will be:

1. Aged ≥ 18 years
2. Graded as mRS 4 or 5 and/or National Institutes of Health Stroke Scale (NIHSS) ≥ 16 (severe or very severe stroke and unable to stand without support/mechanical aid and assistance of two people)
3. Able to give informed consent or assent received from a consultee (see recruitment section)
4. Conscious and responsive to verbal prompts.

#### Exclusion criteria for quantitative methods

Potential participants meeting any of the following criteria will be excluded from trial participation:

1. Systolic blood pressure ≤ 100 mmHg or ≥ 220 mmHg at rest, lying or sitting
2. Oxygen saturation ≤ 87% with or without supplementary oxygen (e.g. severe acute/chronic cardiorespiratory disease)
3. Resting heart rate of ≤ 40 or ≥ 110 beats per minute (e.g. cardiovascular instability)
4. Temperature ≥ 38.5 degrees centigrade or ≤ 35 degrees centigrade
5. Orthopaedic impairments which prevent full weight bearing in standing
6. Malnutrition Universal Screening Tool score of ≥ 2, or deemed to be not meeting nutritional demands for therapeutic interventions by dietician
7. Documented clinical decision for receiving end of life care
8. Unstable coronary or other medical condition that is judged by the Principal/Chief Investigator (PI/CI) or clinical team to impose a medical risk to the patient by involvement in the trial
9. Assessed functionally by specialist clinicians as being a risk to themselves or others due to their inability to follow non-verbal prompts or are behaving erratically
10. Immobile and not weight bearing pre-stroke
11. Additional neurological deficits unrelated to the current or past stroke (e.g. peripheral neuropathy or multiple sclerosis), because these impairments are not related to the condition of interest)
12. Weight of 115 kg or more, this is the weight limit on the standing frames
13. Being discharged out of county, e.g. admitted during holiday/visit to Cornwall or Devon because they would be unable to participate in follow-up assessments
14. If people are registered in another trial, the CI will be contacted to ensure there is no conflict between trials
15. Non-English speaking

#### Inclusion criteria for qualitative methods

##### Patient participants

1. Are able to use a range of communication methods including speech, gesture and/or writing as determined by the ConsentSupport Tool as well as other assessments determined appropriate by SLT based on the SRU, e.g. able to answer the questions in the patient-report outcome measures at the 3-week follow-up assessment visit
2. Able to recall involvement in the study or study processes with or without prompts or aids (e.g. study documentation) as required.

##### Relative participants

1. Aged ≥ 18 years
2. A family member/close friend of a participant registered in the SPIRES feasibility trial
3. Able to provide written informed consent

##### Physiotherapist participants

1. Registered physiotherapist working on the Stroke Rehabilitation Unit for 3 or more days in each week
2. Able to provide written informed consent

### Intervention

The intervention will be delivered in the Stroke Rehabilitation Unit over a period of 3 weeks and will start as early as possible after randomisation to ensure that the treatment can be completed during the participant’s inpatient admission. The functional standing frame programme involves a maximum of 30 min using the standing frame (standing and repeated sit to stand) plus an additional 15 min to provide time for usual care physiotherapy, where participants may practise transfers, upper limb activities or activities chosen by participants or guided by physiotherapists. Participants will be asked to undertake the functional standing frame programme once a day, for a target of a minimum of 5 days per week which is aligned with the RCP Guidelines [34].

The initial frequency and duration of standing may vary according to physical capability as assessed by the treating physiotherapist. The aim is to progress standing time by 30% in each session, up to the maximum of 30 min. Each treating therapist will use their individual clinical reasoning when evaluating participants’ tolerance to standing and ability to tolerate incremental increases in standing duration. The associated Work Instruction (Additional file 1) contains specific details of how to implement this. Standing duration for each session will be recorded by the treating physiotherapist.

Participants will move from sitting to standing with aid of an electronic power lifter and a physiotherapist(s) and assistant as required. Foot sensors (integral to the frame design or customised) will record load through each leg when standing in the frame. The aim is for the foot sensors to be used as biofeedback to encourage equal weight distribution during quiet standing and sit-to-stand. Whilst standing, participants will be supported in the frame with straps at the ankle, knees, hips and trunk (if required). They will be encouraged to undertake progressive task-specific activities:

- Repeated sit-to-stand (aiming for 8–12 repetitions to facilitate strengthening)
- Table top games encouraging postural adjustments and use of upper limbs
- Reductions in postural support, e.g. reducing hip and trunk strap tension during standing, elimination of electronic power lifter for sit-to-stand

Should participants improve to the extent where support from the standing frame is not required; then, unsupported standing/walking can be progressed outside of the frame to optimise physical recovery for the remainder of the 3-week intervention if indicated. Repeated sit to stand (8–12 repetitions) should, however, be continued throughout the 30 min session.

Physiotherapists will record activities undertaken during every session using a Physiotherapy Content Recording Tool (Additional file 2). This checklist is based on the Stroke Physical Therapy Intervention Tool [66] which provides a system for recording physiotherapy treatment for stroke patients and has been modified to reflect current clinical practice in a sub-acute rehabilitation setting. Recording physiotherapy interventions during sub-acute stroke rehabilitation will enable usual physiotherapy management to be defined and inform the subsequent main trial. This will include number of repetitions of sit-to-stand, activities undertaken whilst standing, reductions in postural support, duration of stand and activities undertaken in the 15 min of usual physiotherapy.

For the first three sessions (or longer if deemed appropriate by physiotherapists), blood pressure will be assessed both prior to and during the functional standing frame programme. This is based on the protocol used in the feasibility and safety testing of a very early rehabilitation trial [5]. If, when standing, a participant demonstrates OH (decrease in systolic blood pressure of ≥ 20 mmHg, or a reduction in diastolic blood pressure of ≥ 10 mmHg upon changing body position from a supine position to an upright posture, or sitting to standing) standing will be modified or, if required, ceased for that session. Details are contained in a trial-specific work instruction document.

If the participant demonstrates OH, a protocol to treat OH using non-pharmacological interventions will be implemented, in collaboration with the participant’s ward-based doctor. This protocol will provide details about application and monitoring of a compression garment and continued monitoring of blood pressure (available from corresponding author on request).

To standardise and optimise implementation of the functional standing frame intervention, all treating physiotherapists will be provided with face-to-face trial specific training by the CI and be included on the delegation log, authorised by the CI or PI. They will be provided with a link to the trial website, which will include videos demonstrating standing, examples of how to progress the programme using case scenarios, downloadable schema of suggested task-specific exercises/activities, advice on safety issues and “frequently asked questions”. It will also include trial details (e.g. background, rationale) and CI’s contact details. This will complement a trial-specific work instruction.

### Control (usual physiotherapy)

This is defined as routine physiotherapy stroke rehabilitation for 45 min per day (or as long as tolerated) for a target of a minimum of 5 days per week, which is aligned with RCP Guidelines [34]. There is no agreed upon definition of what constitutes usual physiotherapy; thus, there is likely to be variation in usual physiotherapy due to physiotherapists’ preferences, experience and training. Physiotherapists will record activities undertaken during every session using the Physiotherapy Content Recording Tool (Additional file 2) to enable description of usual physiotherapy across all four Stroke Rehabilitation Units. It will also capture any instances of protocol deviation where physiotherapists implement a standing frame programme with participants in the control group. The protocol, and face-to-face training sessions, advise physiotherapists not to change their usual physiotherapy practice.

It is expected, although not required, that the same physiotherapists will be delivering both interventions (e.g. implementing both the functional standing frame programme and usual physiotherapy). Clear instructions about content of the intervention and control group, combined with fidelity checking, aim to avoid any conflict. However, potential for bias is acknowledged, and therefore, a stepped wedge cluster RCT [28] may be considered for the future main trial. This would allow every site (cluster) to deliver both the control and the intervention, with every cluster switching from control to the intervention, but at different time points.

### Standardisation of the intervention

Treating physiotherapists from each of the three healthcare sites will perform the interventions as part of their clinical role; the CI and a blinded assessor (employed specifically for one site) will undertake the assessments.

**Fig. 2:**
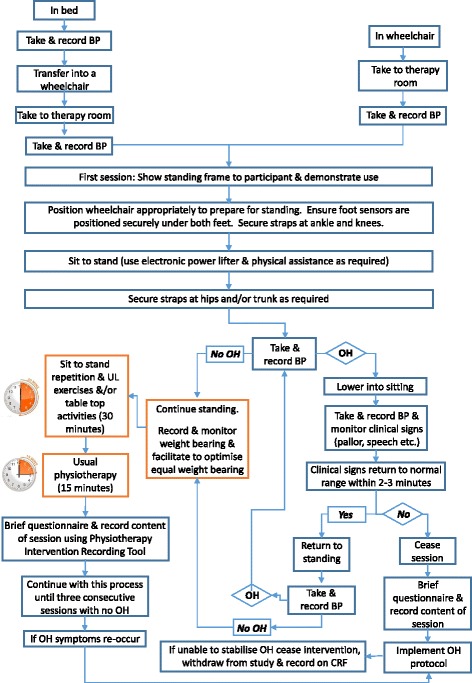
Flow chart 1. Procedure for implementing the functional standing frame programme whilst monitoring BP. This figure represents the procedures for the functional standing frame programme intervention. This first flowchart encompasses the monitoring of blood pressure to ensure participant is safe to continue with the intervention

**Fig. 3:**
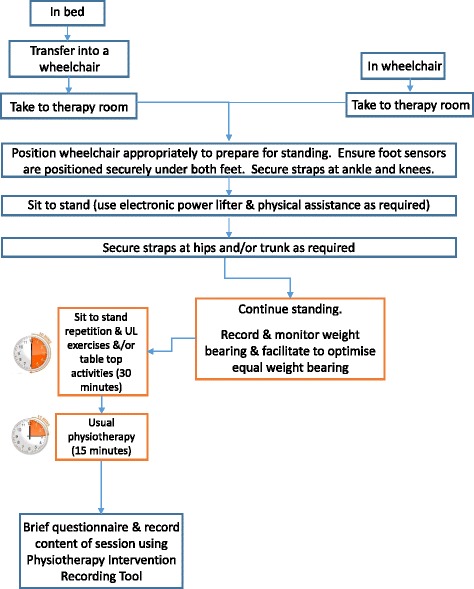
Flowchart 2. Procedure for implementing functional standing frame programme when OH stabilised. This second flowchart provides a diagramatical representation of procedures to follow for the functaional standing frame programme for patient participants who have been deemed to have stable blood pressure

Use of standing frames is incorporated within undergraduate physiotherapy training and is a recognised core skill for neurological physiotherapists. To standardise and optimise implementation of the intervention, treating physiotherapists will receive face-to-face training and an information pack. This includes a written template of what is required to be undertaken within each session (Figs. 2 and 3) and a link to the trial website.

Treating physiotherapists and the assessors are required to record any deviations from the protocol on a Protocol Deviation form.

Inter-rater reliability will be assessed for the blinded assessors on all outcome measures (both primary and secondary).

1. Proposed primary outcome measures

The proposed primary outcome measure assesses functional ability in performing activities of daily living. Functional ability was identified as being important and a priority for people with severe stroke and their relatives during discussions in the development of this feasibility trial.

The Barthel Index of Activities of Daily Living (BI) [41] is frequently used in stroke clinical trials, although was not designed specifically for clinical trials or the stroke population. The BI rates a person’s degree of independence performing functional self-care (feeding, grooming, bathing, etc.) and mobility activities (transferring in/out of bed/chair, walking, etc.). A major limitation of the BI is its floor effect [51], and as a result, it has limited ability to detect change at extremes of ability, making it less discriminating in severe stroke [55].

This feasibility trial provides the opportunity to investigate whether an alternative functional outcome measure is more sensitive to change in people with severe stroke. Therefore, the Edmans Activities of Daily Living Index for Stroke Patients [20] will also be used. This measure has cover all the categories included in the BI; however, the response categories are independent, supervision, help of one and help of two, as opposed to the BI responses which are only dependent or independent for each item assessed.

2) Proposed secondary outcome measures

*Blood pressure in lying and sitting* to determine presence of orthostatic hypotension using sphygmomanometer: Orthostatic hypotension is a decrease in systolic blood pressure of ≥ 20 mmHg, or a reduction in diastolic blood pressure of ≥ 10 mmHg upon changing body position from a supine position to an upright posture, or sitting to standing [39]. This feasibility trial will record the incidence of OH and whether OH limits participation in the functional standing frame intervention.

*Control of trunk* using Trunk Control Test [18]: The Trunk Control Test has demonstrated construct and predictive validity [22] and concurrent validity and interrater reliability [12].

*Knee extension muscle strength* (left and right) using hand held dynamometer [33, 52]: Knee extensor strength is strongly correlated to common daily functional activities such as the ability to sit to stand, stand and walk in people with sub-acute stroke [33]. It is reliable in measuring lower limb strength in people with stroke (ICC = 0.88–0.98) [52].

*Length of hip flexors, hamstrings and ankle plantarflexors* (left and right) using manual universal goniometer [7]: Passive range of movement of these muscles will be measured on both lower limbs. Intra-rater reliability using goniometry to measure ankle plantarflexor length was moderate to good (ICC 0.719–0.892) and inter-rater reliability was moderate (ICC 0.725–0.741) in people with stroke.

*Muscle tone in hip adductors, hamstrings and ankle* (left and right) using Modified Ashworth Scale [24]: The Modified Ashworth Scale shows good inter-rater reliability hip and knee (weighted kappa = 0.82) and ankle (weighted kappa = 0.74); moderate intra-rater hip (weighted kappa = 0.45); good intra-rater reliability for knee (weighted kappa = 0.62) and very good for the ankle plantarflexors (weighted kappa = 0.85) people with stroke [25].

*Mood* using Patient Health Questionnaire (PHQ-9) [72] or Stroke Aphasia Depression Questionnaire-10 (SADQ-10) for participants who have aphasia [61]: PHQ-9 shows good sensitivity (78%) and specificity (96%) for any depression diagnosis regardless of age, gender or ethnicity [72] and the SADQ-10 shows good internal consistency (Cronbach’s alpha = 0.80 and a split-half reliability of *r* = 0.81) [61].

*Health-related quality of life* using Stroke and Aphasia Quality of Life Scale-39 [30] and the EuroQol Group 5 Dimensions, 5 Levels (EQ-5D 5L) [29]: The Stroke and Aphasia Quality of Life Scale-39 has high internal reliability (alpha = 0.74–0.94) and test re-test reliability is also good (ICC = 0.89) [30]. However, it is unknown whether it is feasible for people with severe stroke to use this outcome measure due to communication and cognitive impairments. The EQ-5D-5L shows moderate responsiveness to change (SRM = 0.63) and a minimally clinically important difference of 0.10 [11].

*Fatigue* using a Visual Analogue Scale (Additional file 3) to enable people with aphasia to also rate their level of fatigue [36].

### Qualitative evaluation

Qualitative methodology will enable exploration of the thoughts, feelings and experiences of trial participants, their relatives and treating physiotherapists/PIs. Stratified purposive sampling will be used to achieve maximum variation to request semi-structured interviews with 16 participants (approximately six in the functional standing frame group, six in the usual physiotherapy group and four people who decline to participate or withdraw), eight relatives and eight treating physiotherapists. A combination of face-to-face, telephone or Skype will be offered for the interviews to inform the most appropriate method for the definitive main trial. The aim of the qualitative interviews is to address several uncertainties or unknowns including recruitment, retention, practical implementation of the intervention, acceptability/tolerance of the intervention, informed consent, and suitability and acceptability of outcomes measures to inform the design and implementation of the definitive main trial.

People with mild to moderate aphasia will be interviewed as it is important to seek the perspectives and experiences of people with aphasia due to its high prevalence in people with stroke. However, people with severe aphasia (unable to complete the patient-report outcome measures) will be unable to participate due to inability to comprehend and/or express their views and will therefore be excluded.

All physiotherapists involved in delivering the trial (PIs and treating physiotherapists) will be invited to a focus group at the end of the recruitment period to discuss their experiences of delivering the trial (participant recruitment, documentation, delivery of the intervention, etc.) to further evaluate and improve procedures for the definite trial. All interviews and focus group will be digitally recorded and transcribed verbatim.

### Sample size

As a feasibility trial, a formal sample size calculation based on considerations of power is not appropriate; this trial is not statistically powered to detect between-group clinically meaningful differences in a primary outcome. One of the objectives of this trial is to provide robust estimates of the likely rates of recruitment and follow-up, as well as provide estimates of the variability of the proposed primary and secondary outcomes to inform sample size calculations for the planned definitive trial. There is no consensus on the recommended number of participants required for a feasibility trial, with published “rules of thumb” ranging from 20 to 70 or more participants, when the planned primary outcome is of a continuous nature. A recent paper recommended a feasibility trial sample size should recruit 25 participants per allocated group, if the planned definitive trial will have a two-arm parallel group design, with 90% power and two-sided 5% significance level, to detect a “small” standardised effect size [71]. Therefore, this feasibility trial aims to recruit 50 participants in total.

A target sample size of 50 patient participants will allow the follow-up rate to be estimated to within ± 15%. The follow-up rate at 12 months is estimated to be 70%, which would provide follow-up outcome data on a minimum of 35 participants across both allocated groups and three sites.

### Recruitment

Identification and recruitment will be by a healthcare professional on admission to one of four inpatient Stroke Rehabilitation Unit participating in the trial. This will be supported by the local NIHR Clinical Research Network. The healthcare professionals will log all admissions and screen patients for their stroke severity. All patients classified as a severe stroke will be screened for their eligibility, and any eligible participants will be approached within 48 h of the participant being deemed medically fit for rehabilitation or as soon as practicable.

## Methods: assignment of interventions

### Randomisation and concealment

The randomisation process will follow a strict and auditable protocol. Randomisation will take place after all baseline assessments have been carried out; the blinded assessor will input relevant participant details into the trial website, and randomisation will subsequently be conducted by the Peninsula Clinical Trials Unit (PenCTU) data programmer via the secure web-based system. The allocations will be computer-generated by the PenCTU in conjunction with an independent statistician, in accordance with the PenCTU’s standard operating procedure. The randomisation list and the program that generated it will be stored in a secure network location within the PenCTU, accessible only to those responsible for provision of the randomisation system. This will ensure concealment of both the clinical staff undertaking recruitment and the blinded assessor to each participant’s allocated group.

A minimisation procedure (which has a random element) during the randomisation process will be used to reduce possible imbalance between the two groups (intervention and control). Minimisation factors are:

1. Fatigue scored by the participant (if they are able) at baseline assessment, as measured using a Visual Analogue Scale (VAS) (fatigue (VAS: 4–10) vs. no/minimal fatigue (VAS: 0–3)) (Additional file 3)
2. Presence of OH at baseline, tested using manual sphygmomanometer and a standardised protocol

After randomisation, an automatic email will be sent by PenCTU to the relevant treating physiotherapist to notify them of each participant’s allocated group. Notification that randomisation has taken place (but no details regarding individual participant’s allocated group) will also be sent to the CI. Access to the randomisation code and allocation list will be confined to the CTU data programmer; no one else in the trial team will be aware of participant’s allocated group until formal randomisation is completed, hence maintaining effective concealment.

### Blinding

Trial participants and treating physiotherapists are unable to be blinded due to the nature and complexity of the intervention. However, the assessors undertaking the outcome assessments will be blinded to all participant’s allocated group until after the 3-week assessments have been completed. To undertake the qualitative evaluation, one assessor will be unblinded to group allocation for up to 16 participants. A member of the research team who does not require to be blinded will undertake the stratified purposive maximum-variation sampling.

The initial baseline assessment will be undertaken once consent/assent has been received and prior to randomisation. Every effort will be made throughout the trial to ensure assessments are blinded for the remaining 34 participants whose allocated group will not be unblinded. Treating physiotherapists and/or participants will be reminded not to discuss their allocated group with the assessor during any interaction. At each assessment time point, the blinded assessors will be asked to record if they were unblinded to group allocation, and if so, the reasons for this as well as recording which group they think the participant was allocated to.

## Methods: data collection, management, and analysis

Data will be collected using a range of methods: semi-structured interviews with patient participants, their relatives, and treating physiotherapists/PIs, Case Report Forms and observer completed fidelity checklists.

A) Feasibility outcomes

**Table:** 

Feasibility indicator	Outcome measures	Data collection method
Process		
Recruitment rate	% of participants recruited/time	Case Report Forms
Retention rate	% of participants completed T1, T2, T3, T4, T5	Case Report Forms & Withdrawal Forms
Ability to consent	% of participants consenting	Screening/Approach Logs
Consent rate	% of consultee declarations	Case Report Forms
Eligibility criteria	% of admissions screened & eligible	Screening/Approach Log, Recruitment rate, Interview & Focus Group
Willingness of physiotherapists to recruit	% of admissions screened & approached	Recruitment rate, Interview & Focus Group
Willingness of patients to be randomised	% of participants who refuse to enrol in the trial	Recruitment Rate, Interviews & Focus Group
Acceptability of the intervention	% of withdrawals	Case Report Forms & Interview
Determining usual physiotherapy	Frequency specific physiotherapy interventions are implemented	Case Report Forms
Resources		
Burden	% of participants refusing physiotherapy sessions and follow-up assessments	Case Report Forms & Interviews & focus group
Cost effectiveness	*n* = duration (minutes) of functional standing frame programme session	Case Report Forms & Interviews
Management		
Fidelity	Observe intervention and control group sessions	Fidelity Checklists
Participant adherence	*n* = sessions per week	Case Report Forms
	*n* = minutes in standing	Case Report Forms & interview
	*n* = sit to stand repetitions	Case Report Forms & interview
	*n* = Yes; *n* = No for enjoyment	Case Report Forms & interview
	Score out of 10 for effort	Case Report Forms & interview
	Score out of 10 for fatigue	Case Report Forms & interview
Orthostatic hypotension protocol	% incidence of orthostatic hypotension	Case Report Forms
	% of incomplete sessions due to orthostatic hypotension (OH)	Case Report Forms
Safety		
Intervention	*n* = AE & SAE	Adverse Event and Serious
Data collection	*n* = AE & SAE	Adverse Event Forms

T1: baseline; T2: post-intervention period; T3: 3 months; T4: 6 months; T5: 12 months

B) Clinical outcome measures

Standardised, validated clinician-rated and patient self-reported clinical outcomes will be measured in both groups at baseline, post-intervention (within 1 week) and follow-up (3, 6 and 12 months ± 1 week). Assessments at these time points will be conducted wherever the participant is residing. Due to the average length of stay in inpatient stroke rehabilitation, follow-up assessments at 3, 6 and 12 months are likely to be in the participants’ own home or residential/nursing care facility. All the outcome measures listed below will be undertaken at each of the follow-up trial visits.

### Economic evaluation

As this is a feasibility trial, an economic evaluation will not be carried out. However, the data set for the EQ-5D 5L will be collected to determine if it is feasible to collect these data from people with severe stroke.

### Process evaluation

Process evaluation is a key part of the intervention development process to enable conclusions to be made about the strengths and weaknesses of a trial. This will facilitate decision-making for the definitive main trial. The Medical Research Council guidance [43] recommends process evaluation and highlights the importance of capturing fidelity (whether the intervention was delivered as intended); dose (the quantity of intervention implemented) and reach (whether the intended target population comes into contact with the intervention, and how).

Fidelity will be measured using several mechanisms: (i) treating physiotherapists will record the content of their physiotherapy sessions and adverse events in the Case Report Forms; (ii) an independent assessor will observe one intervention and one control group session at each of the four Stroke Rehabilitation Units at random timepoints during recruitment and complete a fidelity checklist (Additional file 4); (iii) during qualitative interviews with treating physiotherapists.

### Data analysis

A detailed statistical analysis plan will be developed by the lead author and approved by statisticians and the Trial Steering Committee, prior to the final database lock and analyses. The primary descriptive analyses will be on an intention to treat basis. The analyses of the quantitative data will be in two stages, in line with CONSORT guidelines for pilot and feasibility trials [21].

- *Stage 1* will summarise the feasibility outcome data.
- *Stage 2* will summarise the clinical outcome data at each time point. Descriptive statistics of the clinical outcome data will be produced for each trial arm. No formal hypothesis testing will be undertaken of the data.

### Qualitative analysis

The qualitative data will be transcribed verbatim and analysed using thematic analysis [9], using a framework matrix as a data management tool [58, 59].

The qualitative data will be transcripts from one-to-one semi-structured interviews with patients, their relatives and physiotherapists, and the physiotherapist focus group. Anonymised transcribed data will be entered into NVivo software [50]. Narratives generated from the interviews will provide information about the uncertainties and unknowns of this feasibility trial. Interview and focus group participants (patients, relatives and physiotherapists) will be invited to review an initial draft to ensure the analysis represents an accurate overview of participants’ views, experiences and recommendations.

### Safety monitoring

Throughout the trial, all possible precautions will be taken to ensure participant safety and wellbeing. Patient participants will be monitored for adverse events via completion of an aphasia friendly brief interview after every therapy session during the 3-week intervention period. Treating physiotherapists will be asked to report adverse events related to the interventions (e.g. falls, musculoskeletal aches and pains, fatigue) and all serious adverse events (e.g. prolonged/required hospitalisation, life threatening event, death, significant medical event, persistent/significant disability/incapacity) to the research team, regardless as to whether they are thought to be related to the intervention or not and whether they believe these serious adverse events are related to the intervention or not. Additionally, participants will be monitored during their scheduled follow-up visits for adverse events and serious adverse events. Adverse events will be reviewed regularly by the Trial Management Group to determine the relatedness of these events to the intervention. Serious adverse events will be reviewed by the Trial Steering Committee.

### Retention rates and withdrawal

Each participant has the right to withdraw voluntarily from the trial at any time, without any effect on their current or future care. This may be through personal choice (i.e. they withdraw their consent), where it becomes impossible to provide outcome data or comply with any other trial procedures for whatever reason, following consultation/recommendation of a health professional following an adverse event or serious adverse event, or a significant protocol deviation, such as a participant being found to be ineligible post-randomisation. Participants found to be ineligible will be withdrawn. Reasons for withdrawals will be recorded and reported by the treating physiotherapist using a standardised proforma. Participants who wish to stop participating in the functional standing frame group will be asked to remain in the trial for follow-up assessments as per protocol if this is possible, although it is acknowledged that if a participant is receiving end of life care this would not be appropriate.

### Determining progression to the full trial

Progression to a full trial application will occur if minimum success criteria are achieved in key feasibility aims and objectives, and/or if solutions can be identified to overcome any issues. These criteria will be finalised in discussion with the Trial Steering Committee, but are likely to include a minimum of:

- 70% recruitment of the intended 50 patient participants within the 13-month recruitment window
- 80% of consented patient participants randomised to the intervention group engaging with and adhering to a minimum of five session per week during the three-week SPIRES intervention
- 80% completion rate of outcome measures (including follow-up)

### Trial oversight

There are two committees involved in the set-up, management and oversight of this trial: the Trial Management Group (TMG) and Trial Steering Committee (TSC).

The TMG comprises those individuals involved in the development of the protocol and the day-to-day running of the trial. The responsibility of this group is to ensure all practical details of the trial are progressing and that everyone within the trial understands them. This includes monitoring adverse events, recruitment and attrition rates, the project timeline and finances. It will also include responsibility for the release of the trial results and publications. The TMG will meet approximately monthly.

The TSC is responsible for overseeing the conduct of the trial on behalf of the Sponsor and funder, and comprises a group of experienced trialists with majority independent representation including patient and members of the public. The TSC will monitor the scientific integrity of the trial including trial progress, adherence to the protocol and the consideration of new information. The TSC will also be responsible for reviewing accumulating safety data to monitor participant safety. TSC members will be constructively critical of the ongoing trial, but also supportive of its aims and methods.

### Data management, audit and monitoring

The PenCTU will be responsible for data management for the trial. Data will be recorded in trial-specific Case Report Forms by the treating physiotherapists and assessors. Completed forms will be passed to the PenCTU and entered onto a secure web-based database. All data will be double entered and compared for discrepancies. Discrepant data will be verified using the original paper data sheets. Data will be collected and stored in accordance with the Data Protection Act 1998 and will be accessible for the purposes of monitoring or auditing.

### Ethics

The trial will be conducted in accordance with the ethical principles that have their origin in the Declaration of Helsinki, 1996, the principles of Good Clinical Practice, and the Department of Health Research Governance Framework for Health and Social Care, 2005.

All participants (patients, relatives and physiotherapists) will be provided with a Participant Information Sheet approved by HRA and provide written informed consent. A consultee will declare assent if the patient participant lacks capacity to provide informed consent. Physiotherapists will obtain informed consent or assent from participants. The CI will obtain consent for interviews with relatives and physiotherapists.

### Confidentiality

Participants’ anonymity will be maintained on all documents. Data will be collected and stored in accordance with the Data Protection Act 1998 and will be accessible for the purposes of monitoring or auditing. Unique study numbers will be used on all documentation.

### Dissemination

The results of this feasibility trial will inform the design of the anticipated definitive trial, rather than directly inform clinical decision making, since clinical and cost effectiveness cannot be determined at this level. Hence, dissemination, regardless of outcome of this feasibility trial, will focus on publication of the feasibility outcomes, and related methodological issues, in open access peer reviewed journals.

On completion, the full trial report will be accessible on the trial website (https://www.plymouth.ac.uk/research/spires) and via the funding body website, as will the full protocol. This protocol (Version 1.1, dated 28/02/2017) has been written in line with Standard Protocol Items: Recommendations for Interventional Trials (SPIRIT) Guidelines [10]. The Consolidated Standards of Reporting Trials (CONSORT) (Additional file 5) for pilot and feasibility trials [21] and the Template for Intervention Description and Replication (TiDIER) Guidelines [32] (Additional file 6) will be reviewed prior to submitting future publications of the trial results. Authorship of articles will be by the trial team; professional writers will not be used.

Results of this feasibility trial will be presented at national and international conferences, for example UK Stroke Forum, to induce enthusiasm for the potential future trial. Summaries will be posted on to the websites/newsletters of the organisations involved in the recruitment process including Stroke Association and social media platforms such as Twitter to optimise dissemination. In addition, all participants (patients, relatives and physiotherapists) will be offered a lay summary of results and a clinically oriented summary will be provided to recruiting centres. A key output will be an application for funding for a definitive trial, if the results of the feasibility trial meet the criteria for progression.

## Discussion

The importance of early mobilisation for people with severe stroke has been highlighted by people with severe stroke, their relatives and clinicians working in Stroke Rehabilitation Units. This was highlighted during early discussions with patients, their relatives and clinicians where the research topic was strongly endorsed as meaningful and relevant and aligned with patients’ key priorities to “get up and move straight away” after their stroke. This led to the development of the research question and has continued to inform and influence the trial design and remained integral to the development of the methodology, grant application, trial protocol and trial monitoring. The functional standing frame programme has been developed with the aim of addressing this important issue; however, a full evaluation of its effectiveness is essential to inform evidence-based clinical decision-making. Best practice guidance emphasises the need to thoroughly test the feasibility and acceptability of both interventions and trial evaluation procedures prior to undertaking a full-scale assessment of effectiveness [14].

The functional standing frame intervention specifically aims to address the key priorities of patients, their relatives and clinicians, optimising general function, standing and walking. Whilst discussions with people who have had a severe stroke and their relatives identified the importance of people with severe stroke being actively involved in research, they identified several barriers (e.g. fatigue, exercise tolerance, communication difficulties, being physically and psychological overwhelmed and memory difficulties), which are identified and acknowledged in the literature as barriers to recruitment and retention in stroke clinical trials [8]. These barriers have been carefully considered by utilising relevant literature [4] and resources such as Clinical Research Networks and people who have suffered stroke, their relatives and stroke specialist therapists to facilitate inclusion of people with severe stroke. Additionally, the qualitative component will provide the opportunity to explore the impact of the functional standing frame programme intervention, being randomised into the usual physiotherapy group, recruitment and retention.

The usual physiotherapy group provides the opportunity to describe what interventions physiotherapists are currently using to facilitate optimal function after severe stroke, using the Physiotherapy Content Recording Tool. Key clinical guidelines recommend interdisciplinary rehabilitation, of which physiotherapy is primarily aimed at optimising and maintaining activities of daily living, usually starting within the first few days’ post-stroke [34]. There is strong evidence favouring task-specific and high repetitive task-oriented training; however, randomised controlled trials are needed to address which patients may benefit from a specific intervention and at what time post-stroke interventions should be initiated [68]. This underpins the rationale for this feasibility randomised controlled trial to investigate the effects of a functional standing frame programme versus usual physiotherapy in people with severe sub-acute stroke on function, quality of life and neuromuscular impairment.

### Trial status

Recruitment opened in December 2016.

## Additional files

Additional file 1: Work Instruction. (DOCX 19 kb)

Additional file 2: Physiotherapy Content Recording Tool. (DOCX 325 kb)

Additional file 3 Fatigue Visual Analogue Scale. (DOCX 498 kb)

Additional file 4: Fidelity Checklists. (DOCX 82 kb)

Additional file 5: CONSORT 2010 statement: extension to randomised pilot and feasibility trials. (DOCX 23 kb)

Additional file 6: TiDier Checklist. (DOCX 135 kb)

## Abbreviations

ADL: Activities of daily living
AVERT: A Very Early Rehabilitation Trial
BI: Barthel Index
CDRF: Clinical Doctoral Research Fellowship
CI: Chief Investigator
CONSORT: The Consolidated Standards of Reporting Trials
CT: Computer tomography
HRA: Health Research Authority
ICA: Integrated Clinical Academic
ISRCTN: International Standard Randomised Controlled Trial Number
mRS: Modified Rankin Scale
NHS: National Health Service
NIHSS: National Institutes of Health Stroke Scale
NRES: National Research Ethics Scheme
OH: Orthostatic Hypotension
PenCTU: Peninsula Clinical Trials Unit
PI: Principal Investigator
RCP: Royal College of Physicians
RCT: Randomised Controlled Trial
REC: Research Ethics Committee
SPIRES: Standing Practice In Rehabilitation Early after Stroke
SPIRIT: Standard Protocol Items: Recommendations for Interventional Trials
TiDIER: Template for intervention description and replication
TMG: Trial Management Group
TSC: Trial Steering Committee
UK: United Kingdom
VAS: Visual Analogue Scale
