# Supplementary figures and images for: Standing Practice In Rehabilitation Early after Stroke (SPIRES): a functional standing frame programme (prolonged standing and repeated sit to stand) to improve function and quality of life and reduce neuromuscular impairment in people with severe sub-acute stroke—a protocol for a feasibility randomised controlled trial
Source: Pilot Feasibility Stud. 2018 Mar 23;4:66. doi: 10.1186/s40814-018-0254-z (PMC5865293; doi:10.1186/s40814-018-0254-z)

**Additional file 2 Physiotherapy Content Recording Tool**


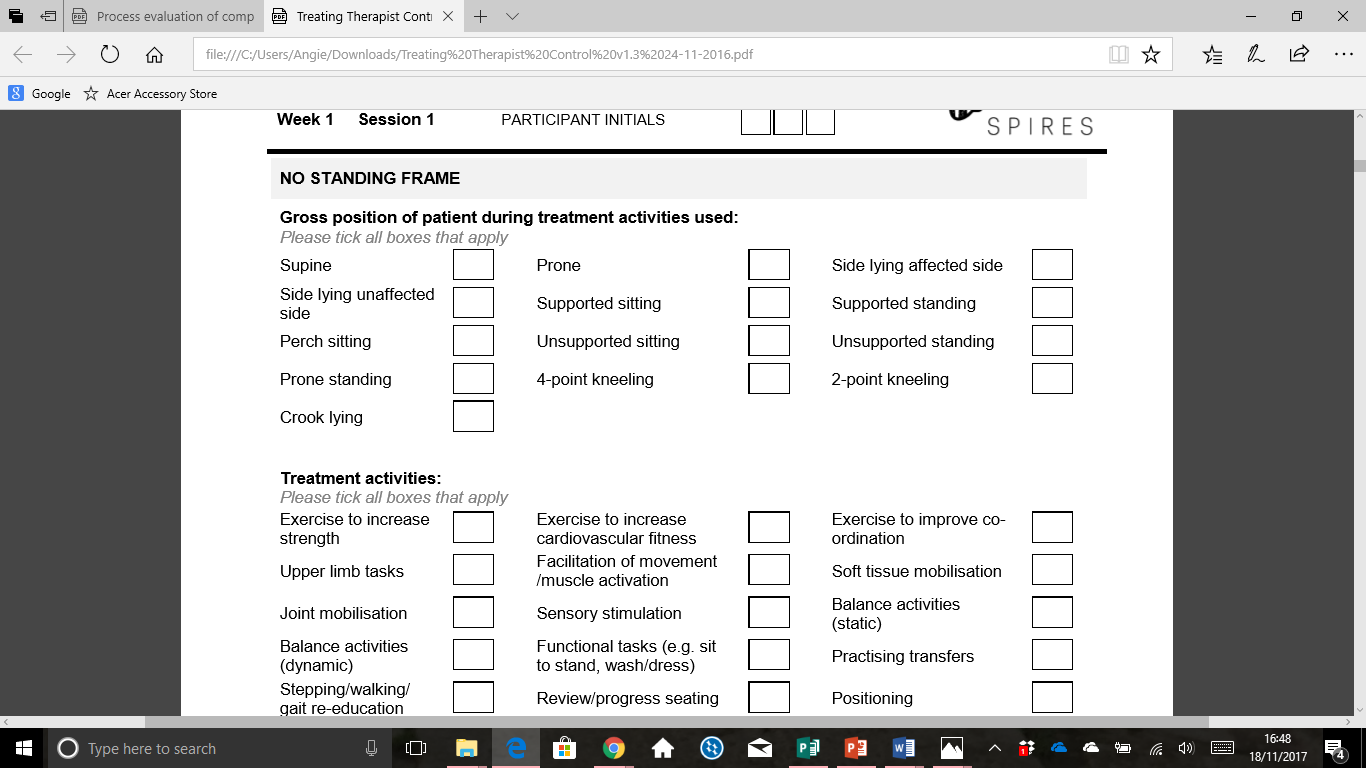


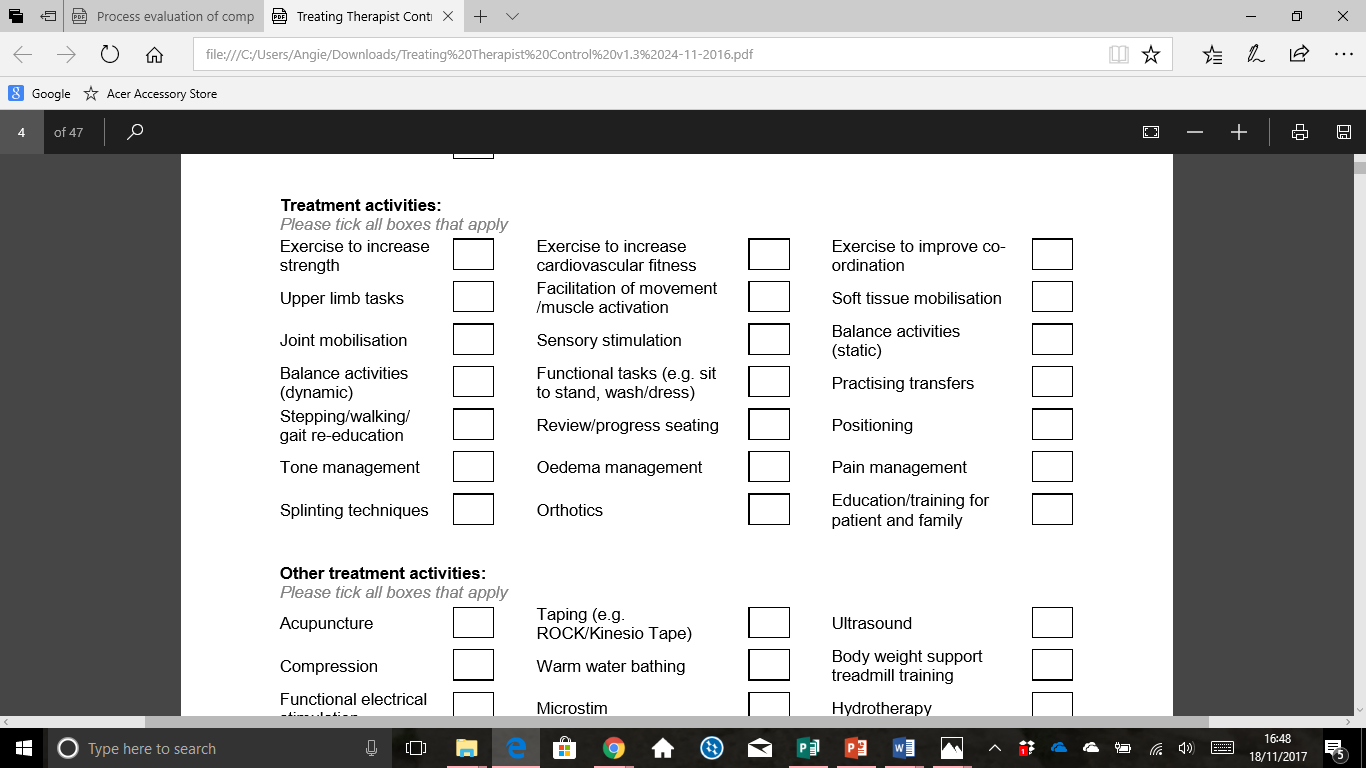


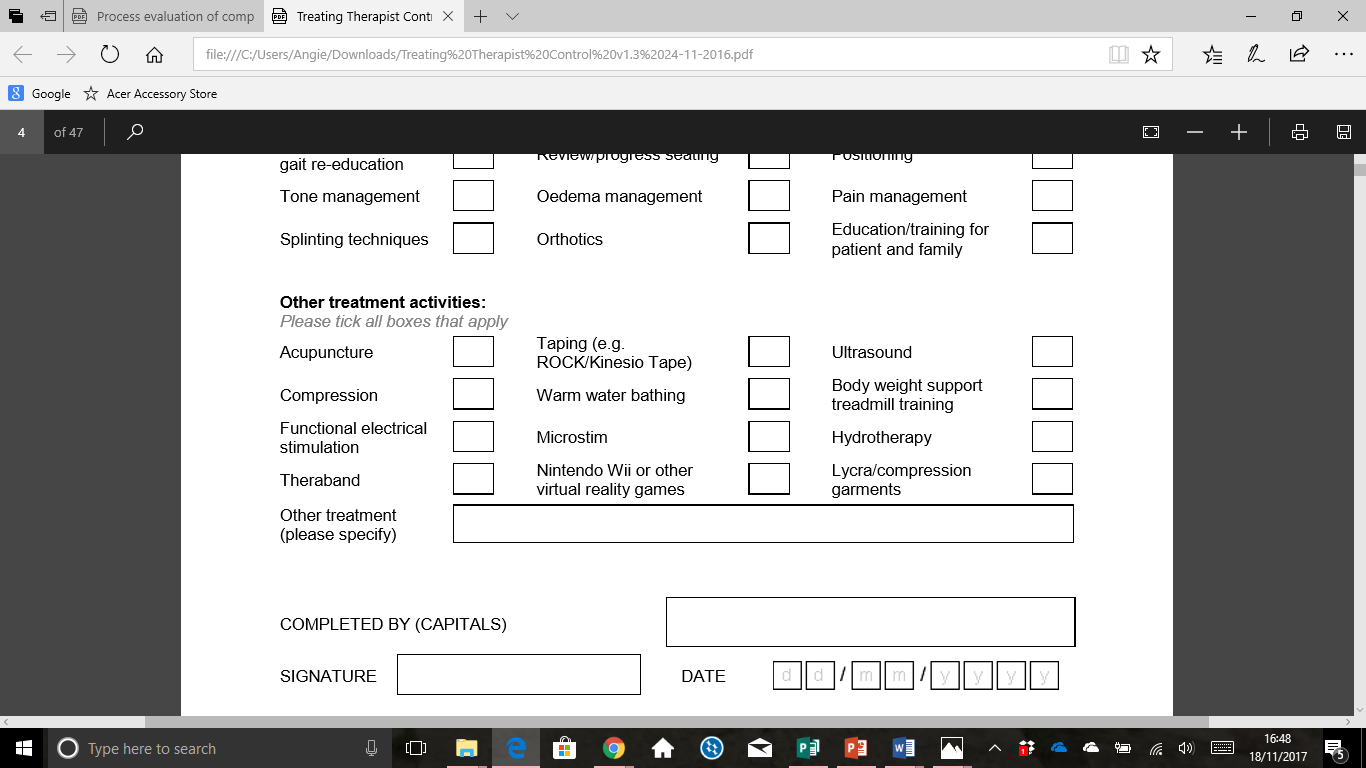

Supplement: Supplementary file 2 — Physiotherapy Content Recording Tool. (DOCX 325 kb) [file 40814_2018_254_MOESM2_ESM.docx]
