# Supplementary material for: Standing Practice In Rehabilitation Early after Stroke (SPIRES): a functional standing frame programme (prolonged standing and repeated sit to stand) to improve function and quality of life and reduce neuromuscular impairment in people with severe sub-acute stroke—a protocol for a feasibility randomised controlled trial
Source: Pilot Feasibility Stud. 2018 Mar 23;4:66. doi: 10.1186/s40814-018-0254-z (PMC5865293; doi:10.1186/s40814-018-0254-z)
Supplement: Supplementary file 3 — Fatigue Visual Analogue Scale. (DOCX 498 kb) [file 40814_2018_254_MOESM3_ESM.docx]

**Additional file 3 Fatigue Visual Analogue Scale**


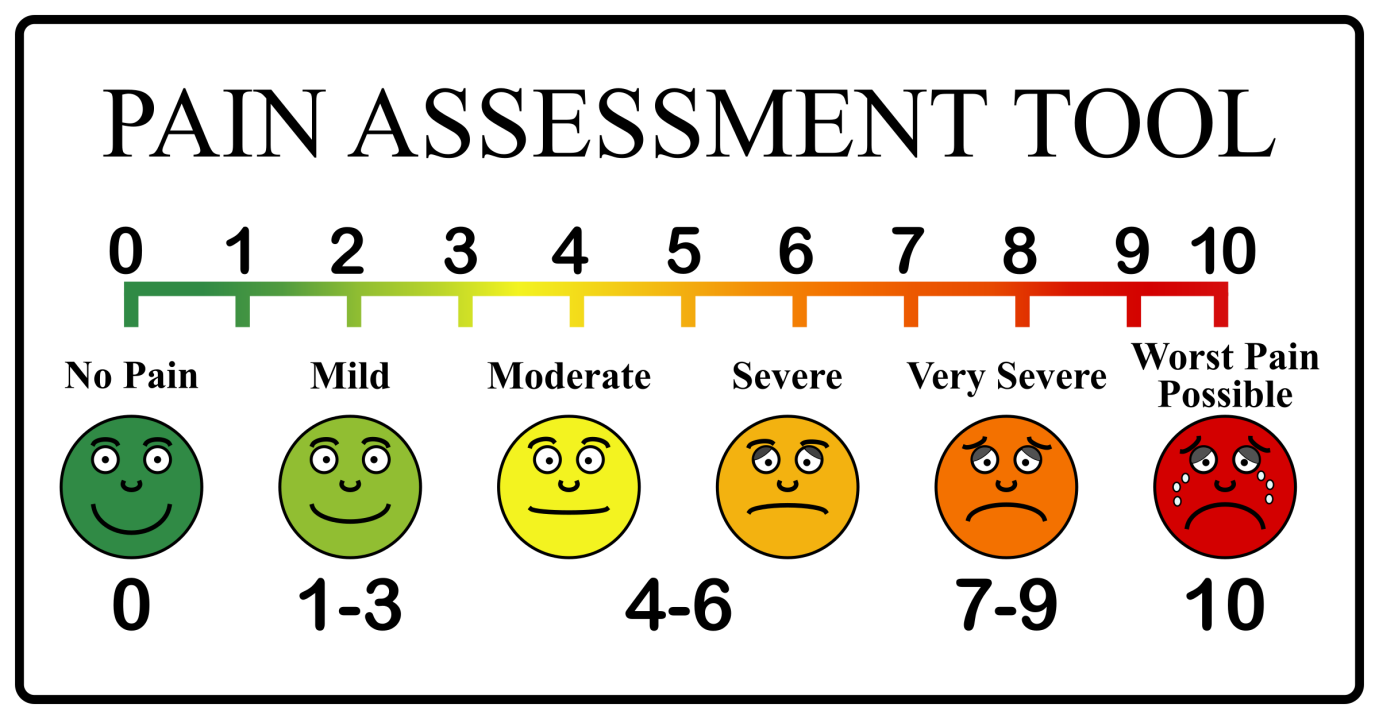


**Not tired at all**

**A little tired**

**Tired**

**Really tired**

**So tired, I can’t do any more**


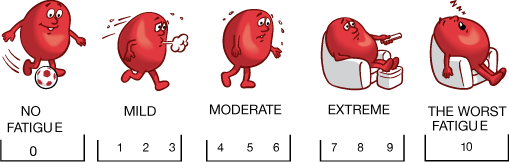

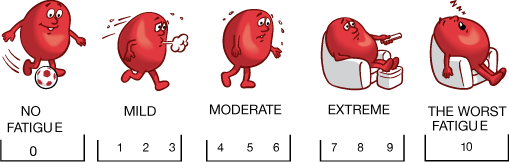

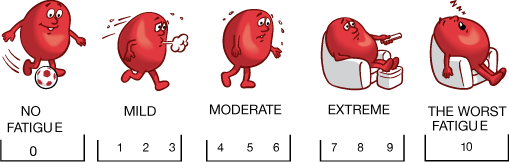

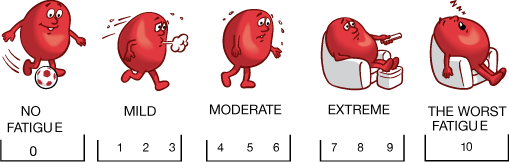

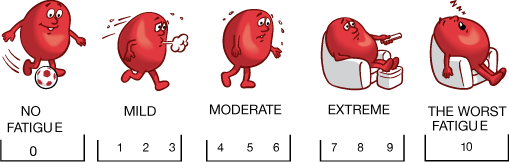


**0**

**1-3**

**4-6**

**7-9**

**10**
