# Supplementary material for: Standing Practice In Rehabilitation Early after Stroke (SPIRES): a functional standing frame programme (prolonged standing and repeated sit to stand) to improve function and quality of life and reduce neuromuscular impairment in people with severe sub-acute stroke—a protocol for a feasibility randomised controlled trial
Source: Pilot Feasibility Stud. 2018 Mar 23;4:66. doi: 10.1186/s40814-018-0254-z (PMC5865293; doi:10.1186/s40814-018-0254-z)
Supplement: Supplementary file 4 — Fidelity Checklists. (DOCX 82 kb) [file 40814_2018_254_MOESM4_ESM.docx]

**
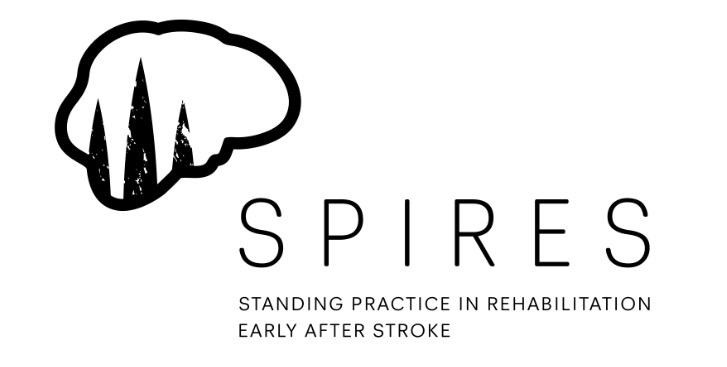
Additional file 4: Fidelity Checklists**

**FIDELITY CHECK LIST FOR INTERVENTION GROUP**

It is important that physiotherapists are following the steps in this checklist when implementing the functional standing frame programme with participants. These steps are detailed in the Work Instruction which accompanies the trial protocol.

Place a 🗸 in the appropriate box on completion of each step and add any comments as appropriate.

| **Participant Number:** **Initials:** | | | | | 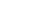  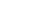  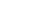 |
| --- | --- | --- | --- | --- | --- |
| **Name of Observer: __________________________________________________________________**  **Name of treating physiotherapist: ______________________________________________________**  **Date of observed session:** _ _ **/** _ _ **/** _ _ | | | | | |
| **ACTION** | **YES** | **NO** |  | **ADDITIONS/COMMENTS/ REASON FOR NOT COMPLETING** | |
| 1. Physiotherapist(s) checks participant’s blood pressure |  |  |  |  | |
| 2. Physiotherapist(s) transfers participant into wheelchair if they are in bed |  |  |  |  | |
| 3. Physiotherapist(s) show participant the standing frame and explain how it works if this is first session |  |  |  |  | |
| 4. Physiotherapist(s) ensure foot sensors are positioned appropriately in the frame |  |  |  |  | |
| 5. Physiotherapist(s) position participant in frame.  If in wheelchair ensure footplates off, brakes on.  If on therapy plinth ensure appropriately supported and plinth brakes on. |  |  |  |  | |
| 6. Physiotherapist(s) adjust knee block/straps to level of tibial tuberosity in sitting |  |  |  |  | |
| 7. Physiotherapist(s) fasten ankle strap |  |  |  |  | |
| 8. Physiotherapist(s) fasten knee strap |  |  |  |  | |
| 9. Physiotherapist(s) position and fasten belt for electronic power lifter |  |  |  |  | |
| 10. Physiotherapist(s) facilitates/assists participant from sitting to standing ensuring hemiplegic upper limb is fully supported |  |  |  |  | |
| 11. Physiotherapist(s) fasten hip/trunk straps as required |  |  |  |  | |
| 12. Physiotherapist(s) check blood pressure if this is this is session 1-3 or if blood pressures have not yet stabilised in sit to stand |  |  |  |  | |
| 13. Physiotherapist(s) facilitates participant to undertake activities in standing |  |  |  |  | |
| 14. Physiotherapist(s) facilitates participant to undertake repeated sit to stand aiming for 8-12 repetitions |  |  |  |  | |
| 15. Physiotherapist(s) explains progression of standing time and repeated sit to stand |  |  |  |  | |
| 16. Physiotherapist(s) follows safe and appropriate procedures for sitting the participant down |  |  |  |  | |
| 17. Physiotherapist records number of sit to stand repetitions in Case Report Form |  |  |  |  | |
| 18. Physiotherapist records duration of standing time in Case Report Form |  |  |  |  | |
| 19. Physiotherapist undertakes brief interview with participant using the aphasia friendly Visual Analogue Scales |  |  |  |  | |
| 20. Physiotherapist explain potential adverse events e.g. muscle stretch pain, fatigue. |  |  |  |  | |
| 20. Physiotherapist(s) undertakes 15 minutes of usual physiotherapy with participant |  |  |  |  | |
| 21. Physiotherapist documents activities undertaken in usual physiotherapy time |  |  |  | | |
| 22. Physiotherapist documents if participant is receiving any treatment of Orthostatic Hypotension |  |  |  | | |
| 23. Physiotherapist documents reasons why participant was unable to take part in today’s session and, if appropriate, records Adverse Events in the relevant section of the Case Report Form. |  |  |  | | |
| 22. Physiotherapist checks for adverse events and records appropriately. |  |  |  | | |

| **Comments**: |
| --- |


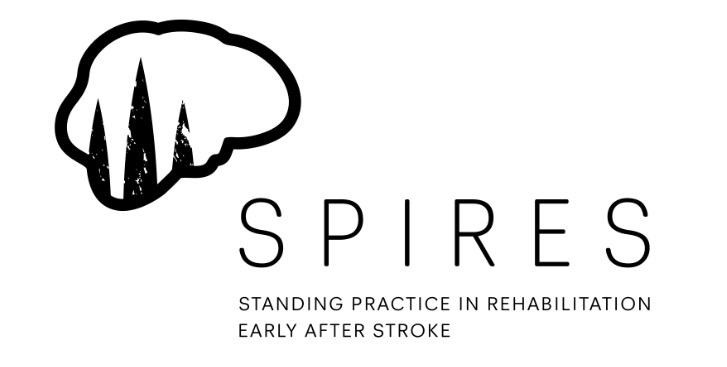


**FIDELITY CHECK LIST FOR CONTROL GROUP**

It is important that physiotherapists are following the protocol and documenting activities undertaken in the usual physiotherapy sessions in the Case Report Forms.

Place a 🗸 in the appropriate box on completion of each step and add any comments as appropriate.

| **Participant Number:** **Initials:** | | | | 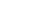  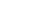  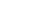 |
| --- | --- | --- | --- | --- |
| **Name of Observer: __________________________________________________________________**  **Name of treating physiotherapist: ______________________________________________________**  **Date of observed session:** _ _ **/** _ _ **/** _ _ | | | | |
| **ACTION** | **YES** | **NO** |  | **ADDITIONS/COMMENTS/ REASON FOR NOT COMPLETING** |
| 1. Physiotherapist(s) undertake usual physiotherapy with participant |  |  |  |  |
| 2. Physiotherapist documents activities undertaken in usual physiotherapy session |  |  |  |  |
| 2. Physiotherapist documents if participant is receiving any treatment of Orthostatic Hypotension |  |  |  |  |
| 3. Physiotherapist documents reasons why participant was unable to take part in today’s session if appropriate and records any adverse events in the Adverse Event section of the Case Report Form. |  |  |  |  |
| 4. Physiotherapist documents duration of session |  |  |  |  |
| 5. Physiotherapists are adhering to the trial protocol and not implementing a standing frame programme |  |  |  |  |

| **Comments:** |
| --- |
