# Supplementary material for: Standing Practice In Rehabilitation Early after Stroke (SPIRES): a functional standing frame programme (prolonged standing and repeated sit to stand) to improve function and quality of life and reduce neuromuscular impairment in people with severe sub-acute stroke—a protocol for a feasibility randomised controlled trial
Source: Pilot Feasibility Stud. 2018 Mar 23;4:66. doi: 10.1186/s40814-018-0254-z (PMC5865293; doi:10.1186/s40814-018-0254-z)
Supplement: Supplementary file 5 — CONSORT 2010 statement: extension to randomised pilot and feasibility trials. (DOCX 23 kb) [file 40814_2018_254_MOESM5_ESM.docx]

# Additional file 5: CONSORT 2010 statement: extension to randomised pilot and feasibility trials


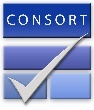
CONSORT 2010 checklist for reporting randomised pilot and feasibility trials

| **Item** | **Standard checklist item** | **Extension for pilot trials** |
| --- | --- | --- |
| Title | Identification of study as randomised | Identification of study as randomised pilot or feasibility trial |
| Trial design | Description of the trial design (eg, parallel, cluster, non-inferiority) | Description of pilot trial design (eg, parallel, cluster) |
| Methods: | | |
| Participants | Eligibility criteria for participants and the settings where the data were collected | Eligibility criteria for participants and the settings where the pilot trial was conducted |
| Interventions | Interventions intended for each group |  |
| Objective | Specific objective or hypothesis | Specific objectives of the pilot trial |
| Outcome | Clearly defined primary outcome for this report | Pre-specified assessment or measurement to address the pilot trial objectives* |
| Randomisation | How participants were allocated to interventions |  |
| Blinding (masking) | Whether or not participants, caregivers, and those assessing the outcomes were blinded to group assignment |  |
| Results: | | |
| Numbers randomised | Number of participants randomised to each group | Number of participants screened and randomised to each group for the pilot trial objectives* |
| Recruitment | Trial status† |  |
| Numbers analysed | Number of participants analysed in each group | Number of participants analysed in each group for the pilot objectives* |
| Outcome | For the primary outcome, a result for each group and the estimated effect size and its precision | Results for the pilot objectives, including any expressions of uncertainty* |
| Harms | Important adverse events or side effects |  |
| Conclusions | General interpretation of the results | General interpretation of the results of pilot trial and their implications for the future definitive trial |
| Trial registration | Registration number and name of trial register | Registration number for pilot trial and name of trial register |
| Funding | Source of funding | Source of funding for pilot trial |
