# Supplementary material for: Standing Practice In Rehabilitation Early after Stroke (SPIRES): a functional standing frame programme (prolonged standing and repeated sit to stand) to improve function and quality of life and reduce neuromuscular impairment in people with severe sub-acute stroke—a protocol for a feasibility randomised controlled trial
Source: Pilot Feasibility Stud. 2018 Mar 23;4:66. doi: 10.1186/s40814-018-0254-z (PMC5865293; doi:10.1186/s40814-018-0254-z)
Supplement: Supplementary file 6 — TiDier Checklist. (DOCX 135 kb) [file 40814_2018_254_MOESM6_ESM.docx]

**Additional file 6: TiDier Checklist**


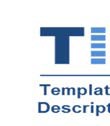

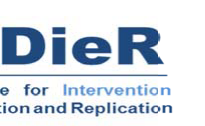


**The TIDieR (Template for Intervention Description and Replication) Checklist*:**


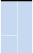
Information to include when describing an intervention and the location of the information

m

| **Item Item Where located **** | | |
| --- | --- | --- |
| **Number** | Primary paper  (page or appendix number) | Other † (details) |
| **BRIEF NAME** |  |  |
| **1.** Provide the name or a phrase that describes the intervention. |  |  |
| **WHY** |  |  |
| **2.** Describe any rationale, theory, or goal of the elements essential to the intervention. |  |  |
| **WHAT** |  |  |
| **3.** Materials: Describe any physical or informational materials used in the intervention, including those |  |  |
| provided to participants or used in intervention delivery or in training of intervention providers. |  |  |
| Provide information on where the materials can be accessed (e.g. online appendix, URL). |  |  |
| **4.** Procedures: Describe each of the procedures, activities, and/or processes used in the intervention, |  |  |
| including any enabling or support activities. |  |  |
| **WHO PROVIDED** |  |  |
| **5.** For each category of intervention provider (e.g. psychologist, nursing assistant), describe their |  |  |
| expertise, background and any specific training given. |  |  |
| **HOW** |  |  |
| **6.** Describe the modes of delivery (e.g. face-to-face or by some other mechanism, such as internet or |  |  |
| telephone) of the intervention and whether it was provided individually or in a group. |  |  |
| **WHERE** |  |  |
| **7.** Describe the type(s) of location(s) where the intervention occurred, including any necessary infrastructure or relevant features |  |  |
|  |  |  |

| **8.** Describe the number of times the intervention was delivered and over what period of time including |  |  |
| --- | --- | --- |
| the number of sessions, their schedule, and their duration, intensity or dose. |  |  |
| **TAILORING** |  |  |
| **9.** If the intervention was planned to be personalised, titrated or adapted, then describe what, why, |  |  |
| when, and how. |  |  |
| **MODIFICATIONS** |  |  |
| **10.ǂ** If the intervention was modified during the course of the study, describe the changes (what, why, |  |  |
| when, and how). |  |  |
| **HOW WELL** |  |  |
| **11.** Planned: If intervention adherence or fidelity was assessed, describe how and by whom, and if any |  |  |
| strategies were used to maintain or improve fidelity, describe them. |  |  |
| **12.ǂ** Actual: If intervention adherence or fidelity was assessed, describe the extent to which the |  |  |
| intervention was delivered as planned. |  |  |

** **Authors** - use N/A if an item is not applicable for the intervention being described. **Reviewers** – use ‘?’ if information about the element is not reported/not sufficiently reported.

† If the information is not provided in the primary paper, give details of where this information is available. This may include locations such as a published protocol or other published papers (provide citation details) or a website (provide the URL).

ǂ If completing the TIDieR checklist for a protocol, these items are not relevant to the protocol and cannot be described until the study is complete.

- We strongly recommend using this checklist in conjunction with the TIDieR guide (see *BMJ* 2014;348:g1687) which contains an explanation and elaboration for each item.
- The focus of TIDieR is on reporting details of the intervention elements (and where relevant, comparison elements) of a study. Other elements and methodological features of studies are covered by other reporting statements and checklists and have not been duplicated as part of the TIDieR checklist. When a **randomised trial** is being reported, the TIDieR checklist should be used in conjunction with the CONSORT statement (see www.consort‐statement.org) as an extension of **Item 5 of the CONSORT 2010 Statement.**

When a **clinical trial protocol** is being reported, the TIDieR checklist should be used in conjunction with the SPIRIT statement as an extension of **Item 11 of the SPIRIT 2013 Statement** (see www.spirit‐statement.org). For alternate study designs, TIDieR can be used in conjunction with the appropriate checklist for that study design (see www.equator‐network.org).
